# Biomechanical validation of novel Nuss procedure simulations for patients with various morphological types of pectus excavatum

**DOI:** 10.3389/fbioe.2023.1297420

**Published:** 2023-11-10

**Authors:** Beop-Yong Lim, Hoseok I, Chiseung Lee

**Affiliations:** ^1^ Department of Biomedical Engineering, Graduate School and University Research Park, Pusan National University, Busan, Republic of Korea; ^2^ Department of Thoracic and Cardiovascular Surgery, School of Medicine, Pusan National University and Biomedical Research Institute, Pusan National University Hospital, Busan, Republic of Korea; ^3^ Department of Biomedical Engineering, School of Medicine, Pusan National University and Biomedical Research Institute, Pusan National University Hospital, Busan, Republic of Korea

**Keywords:** pectus excavatum, Nuss procedure, morphologic types, finite element analysis, computer simulation

## Abstract

A novel Nuss procedure simulation was developed for patients with pectus excavatum considering the displacement of a metal bar and a chest wall model, including the intercostal muscles. However, this simulation was developed for a typical symmetrical patient among the various morphological types of pectus excavatum. Accordingly, this study aimed to validate and confirm the novel simulation for patients with eccentric and imbalanced types, which are severe types of pectus excavatum, considering factors such as depression depth and eccentricity among others. Three-dimensional models of chest walls and metal bars were created for three different types of patients. The rotation-equilibrium displacement and chest wall with intercostal muscles were set according to the methods and conditions of the novel Nuss procedure simulation. The anterior sternal translation and the Haller index derived from the simulation results were compared and verified using medical data from actual postoperative patients. Additionally, maximum equivalent stresses and strains were derived to confirm the suitability of the novel Nuss procedure for each patient type. The severe types had similar precision to the typical type when compared to the actual postoperative patient. Relatively high maximum equivalent stresses and strains were observed on the metal bars and sternum in the severe type, thereby predicting and confirming the biomechanical characteristics of these types. In conclusion, a novel Nuss procedure simulation for severe types was numerically validated. This underscores the importance of biomechanical evaluation through a novel Nuss procedure simulation when planning actual surgeries for severe types of cases.

## 1 Introduction

Patients with congenital pectus excavatum (PEX) require surgical treatment using the Nuss procedure which is performed by a thoracic surgeon. During the Nuss procedure a concave metal bar is inserted and rotated into the chest wall of the patient with PEX. As the concave metal bar rotates and becomes convex, it lifts the sternum. Consequently, the previously collapsed sternum and costal cartilage are repositioned anteriorly, and the chest wall is repaired (Nuss et al., 1998; Nuss and Kelly, 2010). After the Nuss procedure, the repaired shape of the chest wall is analyzed to determine the success of the surgical procedure and the clinical condition of the patient (Dienemann et al., 2014; Saxena, 2017). As the shape of the chest wall after surgery is crucial, it is necessary for thoracic surgeons to predict the postoperative chest wall condition clearly before performing the Nuss procedure. Various studies have been published for this purpose focusing on clinical research by thoracic surgeons regarding the Nuss procedure. However, in recent years there has been a growing body of literature introducing mechanical concepts and methods into the Nuss procedure. One of the applied mechanical concepts and methods is Nuss procedure simulation using the finite element (FE) method. In addition, FE method-based simulations are useful in various biomechanical prediction methods for the surgical treatment of the spine, joints, and blood vessels (Burrowes et al., 2011; Wang et al., 2019; Lim et al., 2020; Nan et al., 2020).

To date, simulation studies of the Nuss procedure have predicted postoperative results by creating a three-dimensional (3D) computerized model that involves anteriorly translating the sternum within a 3D chest wall and metal bar model. The 3D chest wall model consisted of the sternum, ribs, and costal cartilages, and the chest wall tissues were deformed by anterior translation of the metal bar. To improve the accuracy of the chest wall prediction using this simulation methodology, Lim *et al.* (Lim et al., 2023) proposed a novel Nuss procedure simulation study. The main focus of this study was to redefine the displacement of the metal bar and its application to the intercostal muscle (ICM). The Nuss procedure involves inserting a concave metal bar into the chest wall and rotating and equilibrating the bar to shape it into a convex form. Therefore, in the Nuss simulation the condition of the metal bar was redefined by incorporating rotation and equilibrium displacement. In addition, the ICM were integrated into the 3D chest wall model considering their influence on the mechanical deformation of the chest wall. Consequently, the methodology of applying the redefined metal bar displacement method and the 3D chest wall model, incorporating the ICM, in that study showed clinical and mechanical similarities to the patient’s chest wall following the Nuss procedure.

However, Lim *et al.* also have several limitations, and one of the limitations identified within the study is the diversity of morphological types in the PEX. According to Park *et al.*, there are various morphological types of PEX (Park et al., 2004). The classification criteria for these types start by dividing them into symmetrical and asymmetrical. If the centers of the sternum and the depression are co-located, it is symmetrical; otherwise, it is asymmetrical. Among the asymmetric types, the center of the sternum is at the midline. However, the maximum depression occurs on either side of the costal cartilage, which is referred to as an eccentric type. When the center of the depression is at the midline, but one wall of the depression is more severely depressed than the other; it is called the unbalanced type. Several other types of PEX were introduced in this study. In contrast, Lim *et al.* selected and simulated patients with symmetrical PEX, a typical chest wall deformity. It was concluded that the results following the actual Nuss procedure, and the simulation were similar. However, it is necessary to validate and confirm whether the Nuss procedure simulation methodology is suitable for other morphological types, particularly those classified as severe conditions.

PEX is classified into morphological types: 60% are symmetrical and 40% are asymmetrical. Within the asymmetric group, 50% were classified as eccentric, 35% as unbalanced, and 15% as other (Park et al., 2004). Asymmetry in chest wall deformity is a progressive disease with a significant number of cases. The Nuss procedure for asymmetry is considered a challenging surgery (Yoshida et al., 2013; Wang et al., 2022). If the surgery is successful, the patient can lead a normal life without chest wall deformity. However, if the complexity of the deformity is not well understood, it can lead to pericardial, lung and heart damage. Asymmetry significantly affects the feasibility and success of surgery, underscoring the importance of thoracic surgeon performing the Nuss procedure having the necessary surgical experience to deal with asymmetry (Dienemann et al., 2014). Consequently, clinical studies focusing on asymmetric PEX are underway. However, biomechanical studies have mainly concentrated on simulations of symmetric PEX (Chang et al., 2008; Ho Quoc et al., 2013; Rechowicz and McKenzie, 2013; Ghionzoli et al., 2014; Rechowicz et al., 2014; Ye et al., 2017; Sesia et al., 2019; Wang et al., 2022). Further biomechanical studies on severe types are needed, such as the FE method study by Nagasao *et al.*, which showed that the Nuss procedure for asymmetric PEX has a dynamic effect on the spine (Nagasao et al., 2010).

**FIGURE 1:**
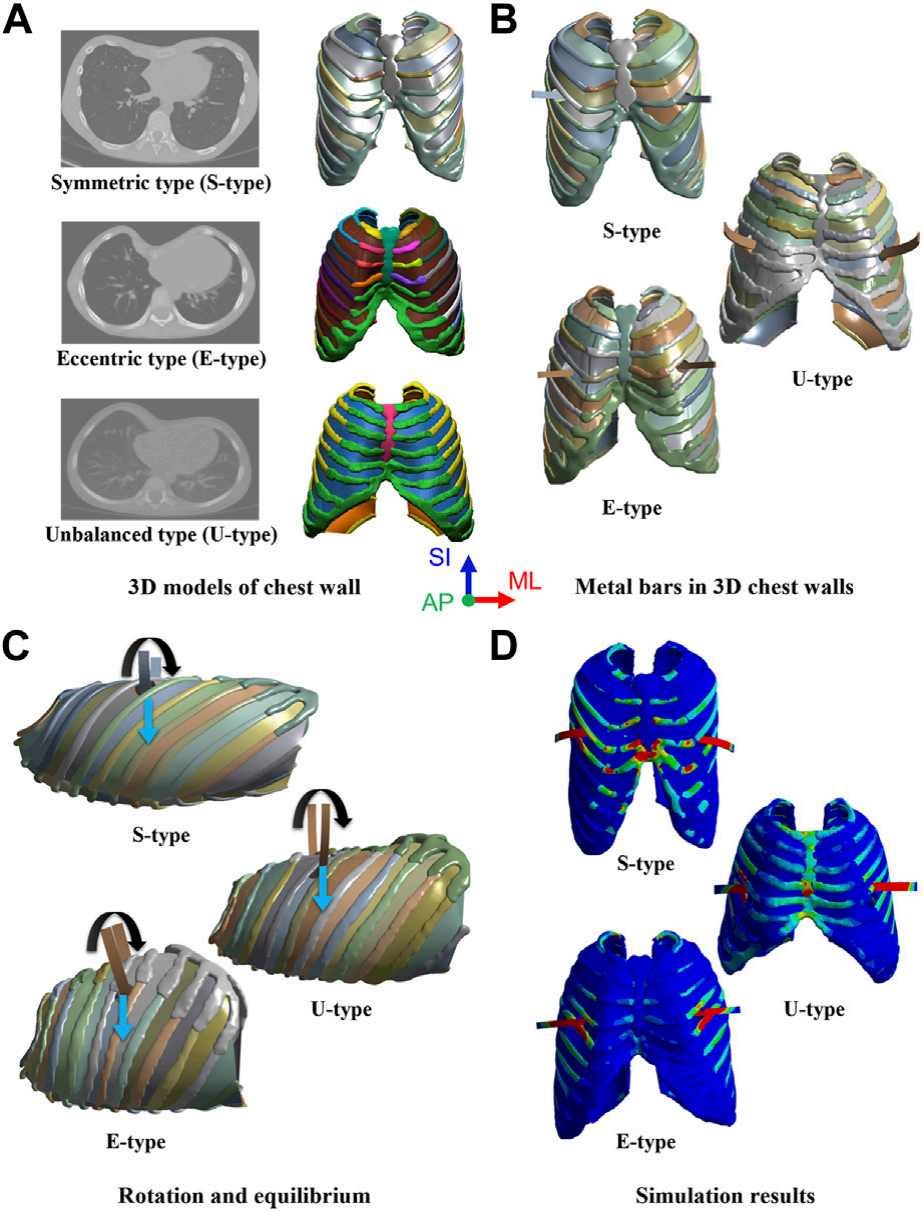
Simulation procedure for morphological types of pectus excavatum (PEX) as **(A)** Three-dimensional (3D) models of the chest wall with morphological types of PEX, **(B)** Inserted metal bars in the 3D chest walls, **(C)** Rotation and equilibrium displacements of the metal bars, and **(D)** Simulation results in scenarios of the morphological types.

Therefore, this study aimed to validate and confirm the various morphological types of PEX by applying a novel Nuss procedure simulation to classify severe types. The major criteria for classifying the PEX types are symmetry, eccentricity, and uniformity. In this study, among the types classified as severe, three were selected based on the above major criteria. The novel Nuss procedure simulation was applied and validated for an already analyzed symmetric patient (S-type), an eccentric patient (A-type) who was asymmetric and had a large difference between the center of the sternum and the center of the depression, and an unbalanced patient (U-type) who was also asymmetric and had a more severe depression on one side than the other. Preoperative 3D models of the chest walls were created for each patient based on the morphological type of PEX. Subsequently, chest wall models, including ICMs, were created similar to the chest wall model of the novel Nuss procedure simulation. The metal bars that were actually applied to each patient were designed in 3D and then inserted into the 3D chest wall models. The conditions for implementing the rotation and equilibrium displacement methods of the metal bar were applied as in the novel Nuss procedure simulation methodology. After performing simulations for each type, the anterior sternal translation, Haller’s index (HI), and maximum equivalent stress and strain on the sternum and metal bar were derived (Figure 1). The results were compared to those obtained after the actual Nuss procedure to confirm their similarity, and the novel Nuss procedure simulation was validated for severe types of PEX.

## 2 Materials and methods

The procedure was performed in the following order: creating 3D chest wall and metal bar models of the patients, inserting material properties for each tissue and matter, applying rotation and equilibrium conditions to the metal bars, and finally performing novel Nuss procedure simulations. The symmetrical, eccentric, and unbalanced morphological types of PEX were chosen as the study subjects, and patients corresponding to each type were selected. Because preoperative 3D chest wall models are created using patients’ medical images, consent is required from selected patients. Because the selected patients where minors consent was obtained from the patients and their parents. The study was conducted with the approval of the Institutional Review Board (IRB No. 1910-017-084).

### 2.1 Fabrication of the 3D chest wall models with PEX patients

FE models of the chest walls and metal bars were created to simulate the Nuss procedure. The 3D chest wall model of the patient was extracted and created from the patient’s medical images. Computed tomography (CT) images of the three types of patients (symmetrical, eccentric, and unbalanced) were obtained and fed into an image processing program (Mimics 23.0, Materialize, Leuven, Belgium). The chest wall tissues (sternum, ribs, and costal cartilage) were masked based on CT images and implemented as a 3D model in the program. ICMs that were not expressed in the CT images were generated by filling the space between the ribs with a shell-shaped rectangular curved surface (Lim et al., 2023). A centerline was created by connecting the centroids at various cross-sections of the rib. After creating a rectangular curved surface encompassing the centerlines of the two adjacent ribs above and below, the average thickness of the ICMs was assigned as the thickness of the curved surfaces (Yoshida et al., 2019). These were defined as ICMs and were generated in the spaces between several adjacent ribs.

**FIGURE 2:**
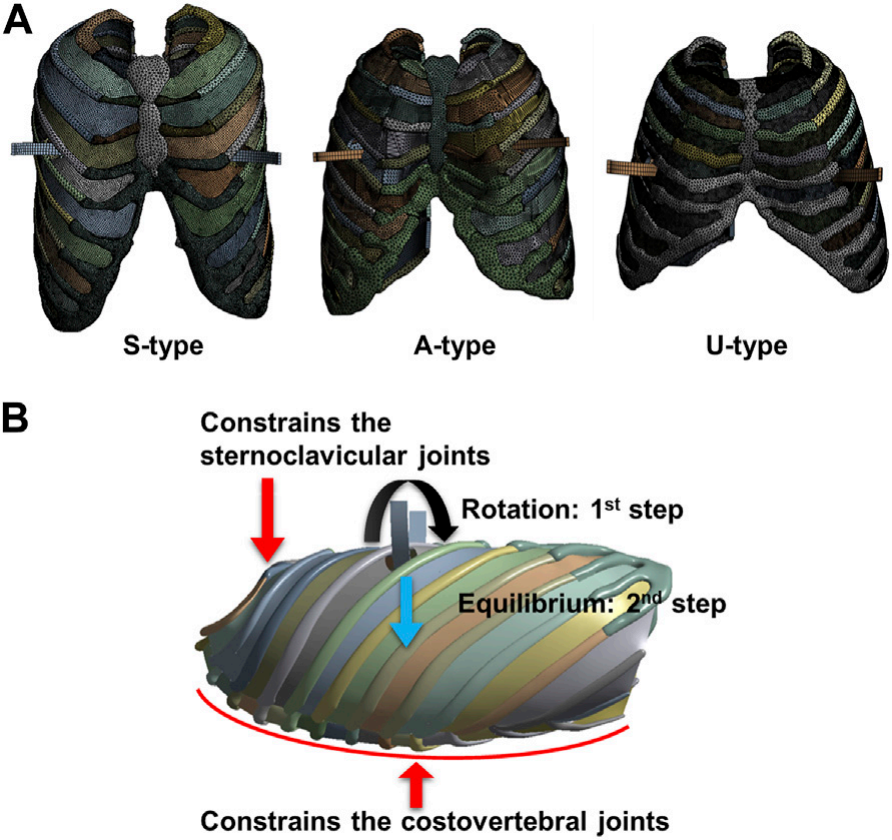
Finite element model and boundary conditions of the chest wall and metal bar. **(A)** Element model of each patient, and **(B)** Boundary conditions for the displacement method of the metal bar and the behavior of the chest wall.

Because the size and shape of each patient’s chest wall differed depending on their age (S: 15, A: 5, and U:4 years old) and PEX type, the applied metal bars also had different shapes for each patient. Additionally, the length and curvature of the metal bar applied to the S-, A-, and U-type patients vary, leading to the customization of the metal bar shape for each individual patient. The length, thickness, and curvature of the metal bar were measured from the CT image of the postoperative patient, and a 3D design was created based on this. The metal bars have different lengths and curvatures, but the thickness is constant at 2.8 mm. Three types of metal bars were modeled using a 3D design program (Inventor 2022; Autodesk, Mill Valley, United States). The designed metal bars were placed in a concave shape and inserted into the constructed 3D chest wall model for each patient. The insertion location of the metal bar was the same as that in the actual surgery and was in the fourth, fourth, and fifth intercostal sections in S-, A-, and U-type patients, respectively. In the actual Nuss procedure, metal bars are inserted into the left and right intercostal spaces on the same line as the maximum depression point of the PEX. Accordingly, the metal bars of each patient were in different intercostal sections. In this 3D chest wall and metal bar model, tetrahedral elements were applied to the chest wall tissues, such as the sternum, ribs, and costal cartilages. The metal bar was a hexahedral element, and the ICMs were a triangular or square element (Figure 2A).

### 2.2 Material properties for the chest wall and metal bar

A patient’s chest wall is composed of the sternum, ribs, costal cartilage, and ICMs. The results of physical quantities such as deformation, stress, and strain of the chest wall vary depending on the material characteristics of each tissue. Therefore, the material properties of the sternum, ribs, costal cartilage, ICMs, and metal bars were incorporated into the 3D model. The interior of the sternum and ribs is divided into cortical and cancellous bones, which have different material properties. Material properties were applied to the cortical and cancellous bones of the sternum and ribs, respectively. Additionally, in the 3D chest wall model, the sternum and ribs should be divided into cortical bone and cancellous bone. Accordingly, a method of covering the surface of the existing sternum and ribs with a virtual shell model with a thickness was adopted. The surface coating function was used on the surfaces of the sternum and ribs to represent cortical bone. The average cortical bone thickness of the sternum was applied, and different cortical bone thicknesses of the ribs were applied depending on their location (Lynch, 2015; Lim et al., 2020). Deformation of the chest wall and metal bar following the Nuss procedure was observed to maintain the plasticity or fracture resistance. Accordingly, the chest wall and metal bar were assumed to exhibit material behavior in the elastic section, and were defined as linear and isotropic materials (Table 1).

**TABLE 1:** Material properties for chest wall and metal bar.

Materials		Young’s modulus (MPa)	Poisson’s ratio	References
Sternum	(Cortical bone)	11,500	0.3	Furusu et al. (2001)
	(Cancellous bone)	40	0.45	Furusu et al. (2001)
Ribs	(Cortical bone)	5,000	0.3	Furusu et al. (2001)
	(Cancellous bone)	40	0.45	Furusu et al. (2001)
Costal cartilage		37.5	0.3	Xie et al. (2017)
Intercostal muscle (ICM)		10.3	0.3	Wang et al. (2019)
Metal bar (Titanium)		105,000	0.3	Zhong et al. (2014)

### 2.3 Boundary conditions for the novel nuss procedure simulation

The displacement of the metal bar was configured to incorporate both rotation and equilibrium displacement in the novel Nuss simulation methodology. In this study, simulation of each type of metal bar involved two steps, first setting the rotation (first step) and then the equilibrium (second step) displacement conditions within the chest wall. As the concave metal bar was placed within the chest wall, it was rotated 180 °and placed in a convex shape. The rotation axis of the metal bar was designated as a straight line connecting the left and right intercostal spaces where the metal bar was inserted. The metal bar was then rotated and swept anteriorly to translate the sternum. Therefore, the contact condition between the sternum and metal bar was set to be frictionless. In the actual Nuss procedure, a small degree of translation appeared on the chest wall after the metal bar was rotated. To implement the equilibrium displacement, the metal bar that had completed the rotation was freed to translate in the posterior direction of the chest wall (Figure 2B). After the metal bar was rotated, the anteroposterior displacement condition of the metal bar was released. When the condition is released, the metal bar is free to displace in the anteroposterior direction. The displacement in the posterior direction was expressed naturally.

Thoracic surgeons performing the Nuss procedure swap or translate metal bar to accurately correct the PEX. In this study, the displacement of the metal bar was carried out as rotation displacement as the first step, and equilibrium displacement as the second step. At this time, the rotation displacement of the first step was simulated more accurately by rotating and slightly translating the metal bar in the anterior or superior direction of the sternum. This translation was also performed according to the actual thoracic surgeon’s correction method. However, since rotation is the main displacement, the first step was named rotation displacement. In conclusion, the rotation displacement of the first step can be said to be the rotation of a metal bar that includes some translations.

The chest wall controls anterior translation of the sternum by imposing several constraints when the sternum is affected by a metal bar. Representative constraints include controlling the chest wall behavior of the sternoclavicular and costovertebral joints. The sternoclavicular joints are the areas between the clavicle and sternum that restricts the anteroposterior translation of the manubrium, the upper part of the sternum (Lim et al., 2020). Accordingly, conditions were set to constrain anteroposterior translation in the sternoclavicular joint region of the 3D chest wall model. The costovertebral joints are the areas between the vertebrae and the ribs that constrain the translation of the posterior ribs. Therefore, translation of the costovertebral joints posterior to the ribs was constrained under fixed conditions (Figure 2B). In addition, boundary conditions between the chest wall tissues such as the sternum, ribs, costal cartilages, and ICMs were required. Each adjacent tissue is tightly coupled without being separated during chest wall behavior by the Nuss procedure. Accordingly, bonded conditions were set in which adjacent tissues are attached to each other without being separated.

## 3 Results

FE method-based simulations were performed according to the scenarios for symmetrical, eccentric, and unbalanced morphological types. All the series were analyzed using an FE analysis program (ANSYS 2022 R2, Ansys Inc., Canonsburg, United States). The computer processor used in the simulation was an AMD Ryzen Threadripper 3990X with 2.90 GHz, 64-core, and 256 GB of RAM. The amount of anterior sternal translation, HI, equivalent stress, and strain on the sternum and metal bar were analyzed as indicators to validate the novel Nuss procedure simulation. The amount of anterior sternal translation and HI were compared with the actual measurements of each patient, the equivalent stress and strain were compared by type, and the mechanical characteristics were analyzed.

### 3.1 Anterior sternal translation

**FIGURE 3:**
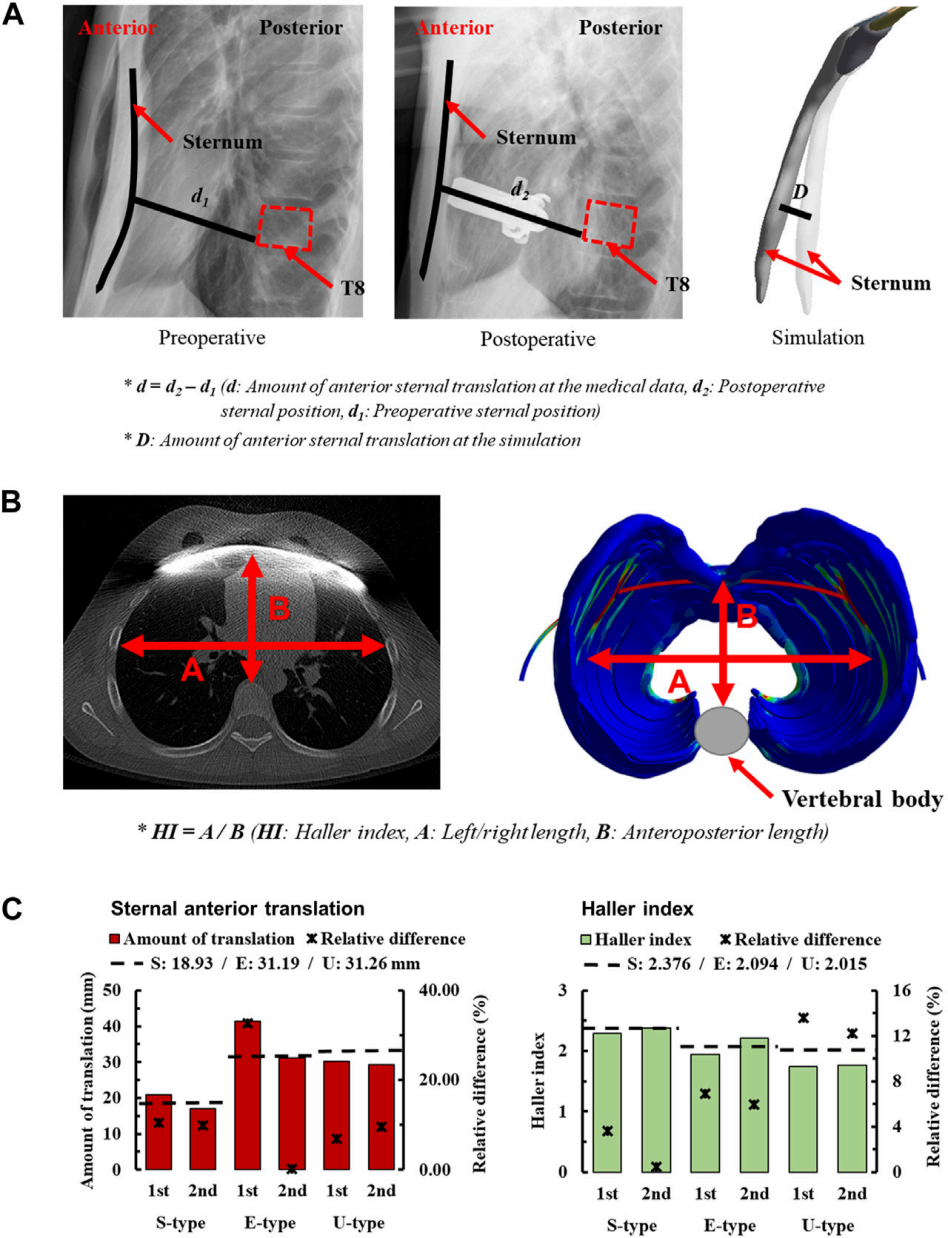
Amount of sternal anterior translation for morphologic types of PEX; **(A)** Measuring the amount of sternal anterior translation using medical images, **(B)** Method of measuring the Haller index (HI) using medical images **(C)** Sternal anterior translation and HI of morphologic types for each scenario.

In the study of the novel Nuss procedure simulation, the accuracy of the methodology was assessed by quantifying the anterior sternal translation. To validate the methodology, the amount of anterior sternal translation was measured in patients with S-, E−, and U-types. The amount of anterior sternal translation in actual patients was determined by measuring the distance of sternal movement from X-ray images. Pre-and postoperative radiographs were obtained to confirm the sagittal axis of the chest wall. A straight line was created starting from the point where the sternum touched the metal bar and ending at the point where it touched the vertebrae in the postoperative image. This straight line was parallel to the metal bar, and the length of the straight line d_2_ was measured. The position of the straight line connecting the sternum and vertebrae was directly substituted into the preoperative radiographs. Another straight line was created in the preoperative image, and the length of this straight line, d_1_, was measured. The difference between the lengths of the two straight lines, d, was defined as the amount of anterior sternal translation, and was compared with the simulation. The amount of anterior sternal translation in the simulation was derived by measuring the difference in sternal deformation D before and after the FE analysis (Figure 3A).

In the simulations, the amount of anterior sternal translation was derived for each scenario considering S-, E−, and U-type patients and stages 1 and 2 according to the displacement of the metal bar. The concept of relative difference was introduced to numerically express the difference between the measured anterior sternal translations of actual patients and the simulation results. When checking the relative difference between each scenario, the first step of E-type was the largest difference from the actual patient at 32.67%. In contrast, the second step of the E-type was the closest result to that of the actual patient at 0.22% (Figure 3C). The relative differences between the S- and U-types were mostly less than or within the 10% range. There were no significant differences in the amount of anterior sternal translation in actual patients, and the results were considered relatively close to those of actual patients.

### 3.2 Haller index (HI)

The second quantitative indicator chosen to assess the accuracy of the methodology in the novel Nuss procedure simulation is the HI. It is an indicator of the cross-section of the chest wall at the point of maximum depression in patients with PEX. It involves measuring both the left-right (transverse) and anteroposterior lengths of the space inside the chest wall from a cross-sectional image. The left-right (transverse) length was measured at the point where the left and right ribs were the farthest from the inside of the chest wall, representing the maximum distance between the ribs. Anteroposterior length is the distance from the vertebra anteriorly to the sternum or costal cartilage anteriorly at the point of depression. The minimum distance from the vertebra to the sternum or costal cartilage was also measured. The left-right and anteroposterior lengths of the chest wall cross-section are measured, and the HI is calculated by dividing the left-right (transverse) length of the chest by the anteroposterior length (Daunt et al., 2004). Cross-sections of the chest wall at the point of maximum depression were confirmed in preoperative and postoperative CT images of the S-, E−, and U-types. The left-right and anteroposterior lengths were measured in each patient’s chest wall cross-section, and preoperative/operative HIs were calculated for each patient (Figure 3B).

The HIs were measured before and after the simulations were performed for each scenario, considering the S-, E−, and U-types of patients and metal bar displacements. In addition, relative differences were introduced and calculated to numerically express the difference between the calculated HI of actual patients and simulations. When checking the relative difference between the HI of the actual patient and that of the scenario, the first step of the U-type had the largest difference from the actual patient at 13.63%. In contrast, the second step of the S-type was the closest result to that of the actual patient, at 0.63% (Figure 3C). The relative differences between patient’s first and second steps were similar. In addition, it was confirmed that the relative difference gradually increased in the order of S-, E−, and U-types.

### 3.3 Maximum equivalent stress and strain on sternums and metal bars

To quantify and schematize the mechanical effects of the S-, E−, and U-types owing to the Nuss procedure, the maximum values and distribution patterns for the equivalent stress and strain should be confirmed in the simulation results of each patient. The equivalent stresses and strains on the sternum and metal bars after the simulations were confirmed using the same method as that in the novel Nuss procedure simulation. The maximum equivalent stress and strain values were derived from the results corresponding to each type of patient scenario and metal bar displacement. The overall distributions of stress and strain on the sternum and metal bar were schematized. Based on the mechanical results, the internal strength and deformation of the sternum and metal bar were predicted for each patient.

**FIGURE 4:**
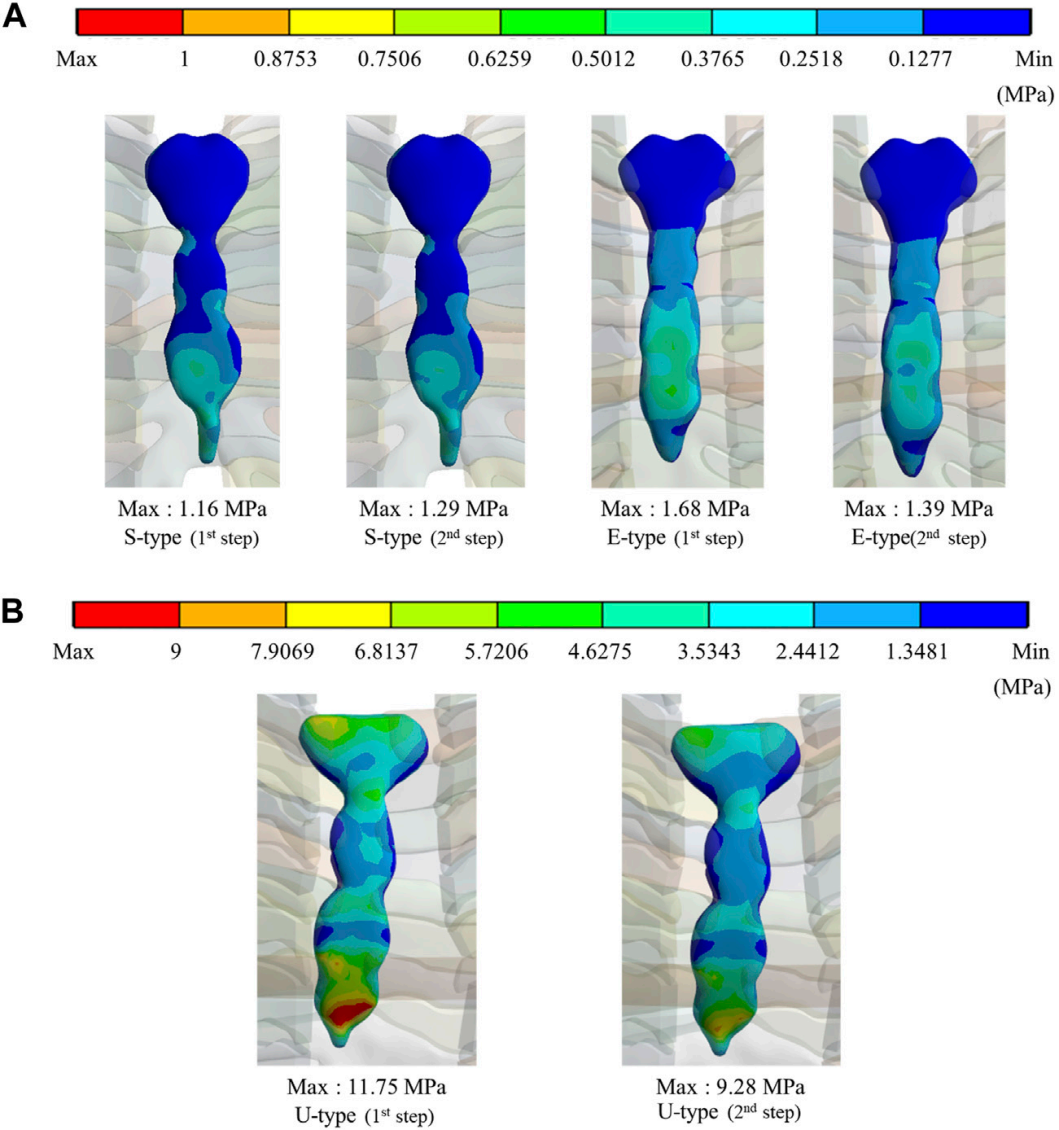
Maximum equivalent stresses and distributions on the sternum for **(A)** Maximum equivalent stresses and distributions of S- and E-types, **(B)** Maximum equivalent stresses and distributions of the U-type.

**FIGURE 5:**
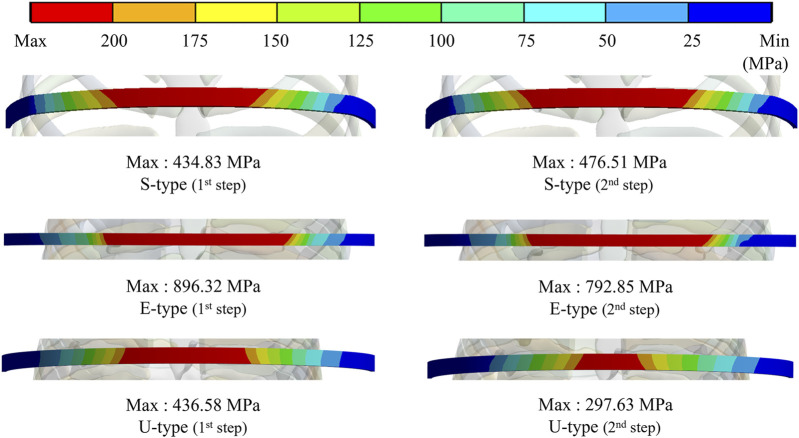
Maximum equivalent stresses and distributions on the metal bar of S-, E-, and U-types.

To assess the degree of strength applied to the inside of the sternum and metal bar using the Nuss procedure, the maximum values and distribution patterns of the equivalent stress were confirmed. The distributions of equivalent stress on the sternum showed that, overall, high internal strengths were distributed to the sternum in the following order: S-, E−, and U-types. Overall, higher internal strengths was observed in the U-type, and the maximum equivalent stress was also the highest. For the S- and E-types, the maximum equivalent stresses ranged between 1 MPa and 2 MPa. However, For U-type, the maximum equivalent stress was 11.75 MPa in the first step and 9.28 MPa in the second step. This implies that the U-type had a maximum equivalent stress that was approximately 10 times that of the other types (Figures 4,8A). The distribution of the equivalent stress on the metal bar exhibited a similar pattern for all types, with the stresses distributed from the center of the metal bar in contact with the sternum. The maximum equivalent stresses on the metal bar occurred in the E-type at 896.32 and 792.85 MPa, which were higher than those in the other types. However, it was confirmed that the maximum equivalent stress was lowest in the second step of the U-type (Figures 5,8A).

**FIGURE 6:**
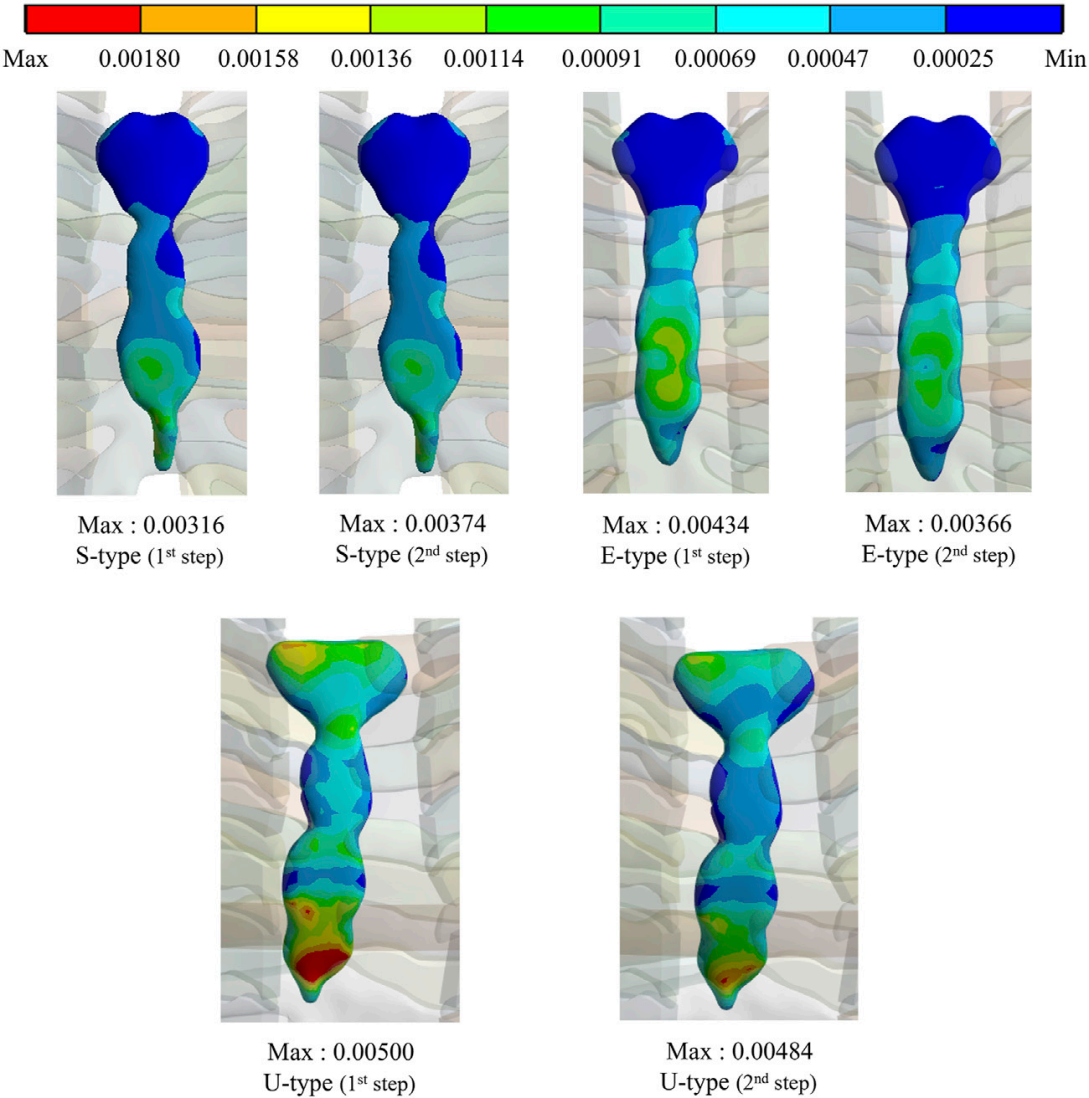
Maximum equivalent strains and distributions on the sternum of S-, E-, and U-types.

**FIGURE 7:**
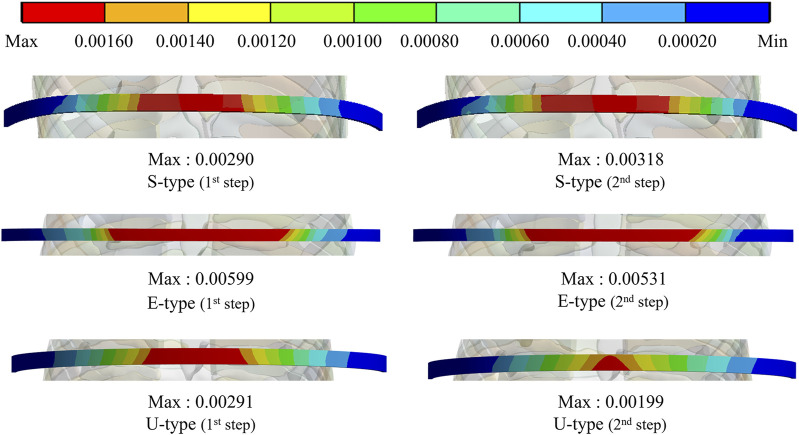
Maximum equivalent strains and distributions on the metal bar of S-, E-, and U-types.

**FIGURE 8:**
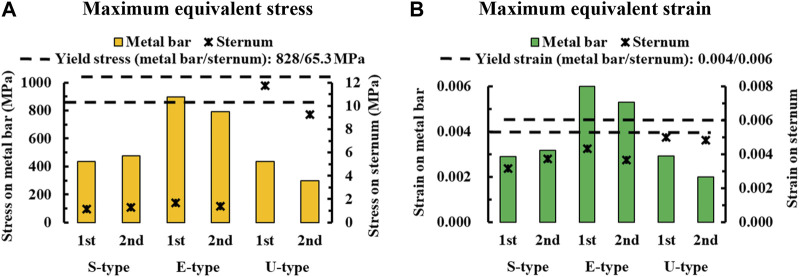
Numerical comparisons of maximum equivalent stress and strain according to S-, E−, and U-types for **(A)** Maximum equivalent stresses on the sternum and metal bar, **(B)** Maximum equivalent strains on the sternum and metal bar.

The maximum values and distribution patterns of the equivalent strain were confirmed to determine the degree of deformation of the sternum and metal bar using the Nuss procedure. The equivalent strains on the sternum were distributed in the same order as the stress, with high strains distributed in the order of S-, E-, and U-types. More deformation occurred in the U-type sternum, and the maximum equivalent strain was the highest. The S- and E-types exhibited similar maximum equivalent strains. However, the U-type had slightly higher maximum equivalent strains than the other types at 0.00500 and 0.00484 (Figures 6,8B. The distributions of the equivalent strain on the metal bar were similar to those of the equivalent stresses, and it was confirmed that the strain increased toward the center of the metal bar for all types. The maximum equivalent strains of the E-type on the metal bar were 0.00599 and 0.00531, respectively, which were higher than those of the other types. Other types were distributed at similar levels, between 0.00190 and 0.00320 (Figures 7,8B).

## 4 Discussion

This study aimed to biomechanically validate the novel Nuss procedure simulation methodology in patients with various morphological types of PEX. In the novel Nuss procedure simulation, when the chest wall is corrected using a metal bar it does not simply move anteriorly but also undergoes rotational and equilibrium displacements. This methodology was applied to patients with various morphological types of PEX, particularly those with severe PEX, to validate the novel Nuss procedure simulation. Among the various morphological types, symmetrical, eccentric, and unbalanced types were selected, and 3D models of the patients’ chest walls were created. After inserting the 3D metal bar into the chest wall of each patient, the rotation and equilibrium displacements were applied as boundary conditions for the metal bar. Nuss procedure simulations were performed according to the patient type and displacement of the metal bar. Consequently, the study determined the amount of anterior sternal translation, HI, equivalent stress, and strain on the sternum and metal bar. This study was conducted to validate the novel Nuss procedure simulation by comparing postoperative results of patient and the results of different scenarios.

The first validation variable of the Nuss procedure simulation was the amount of anterior sternal translation in real patients and the simulation results. In actual postoperative S-type case the magnitude of anterior sternal translation measured 18.93 mm. The simulation results indicated that the relative difference between the first and second step was approximately 10%. The relative differences between the first and second step of the U-type were 6.85% and 9.59%, respectively, which were similar to or lower than those of the S-type. However, there was a significant difference between the first and second step of the E-type, and the second step was very low (0.22%). Therefore, the results of the remaining types, except for the first step of type E, demonstrated satisfactory validation results in terms of the amount of anterior sternal translation. In particular, the second step of E-type provided a strong validation for the novel Nuss procedure simulation.

The second validation variable was analyzed using the HI calculated from the actual patient data and simulation results. The HI for the second step of the S-type procedure was closest to that of the actual postoperative patient, with a relative difference of 0.46%. The relative difference for the E-type was approximately 6%–7%, and that for the U-type was 12%–13%, which was larger than that of the S-type. However, all relative differences remained under 10% and demonstrated consistency with actual patient data validating the simulation results. The relative difference in the second step for the E-and U-types was slightly lower than that in the first step. However, the relative difference in the second step for the S-type was lower. The relative difference in the second step was low for all types, especially for the S-type, and the rotation and equilibrium displacements were considered more accurate. The overall relative differences in the HI tended to be low for all types, and the second step was lower for each type. This validation further supports the use of the novel Nuss procedure simulation.

The final variable was validated by analyzing the equivalent stress and strain on the sternum and metal bars of each type. The distribution pattern and maximum values of the equivalent stress and strain on the sternum tended to differ for the U-type. As the distributions of the equivalent stress and strain were concentrated in the lower part of the sternal body and xiphoid, the U-type was significantly influenced by the mechanics of the Nuss procedure. This was due to the degree of imbalance between the left and right sides of the chest wall, which was more pronounced in the lower part of the sternum, where the maximum depression was observed. In particular, the maximum equivalent stresses of the U-type were 9–12 MPa, approximately 10 times greater than those of the other types. Because this did not reach the yield stress of the sternum, it was concluded that there was no problem with sternal plasticity (Jansová et al., 2015). The maximum equivalent stress and strain for each type of metal bar indicated that the E-type bar exhibited higher values than the other types. Because the values were approximately twice as high as those of the other types, it was predicted that the load on the metal bar for the E-type was considerably large. This was because the point of maximum depression was not centered on the sternum and the main load on the metal bar was eccentric. This eccentric load resulted in the high internal strength of the metal bar, which was slightly higher than its yield stress and strain of the metal bar (Standard, 2011). Because the equivalent stresses and strains of the different types of metal bars tended to fall within the yield stress range, it was determined that there were no specific issues related to plasticity.

To validate the novel Nuss procedure simulation methodology, simulations were conducted for patients with severe types of PEX. Among the various types of PEX, eccentric and unbalanced types were selected. However, there were also long canals and combinations of canal types. Therefore, a study is required to validate novel Nuss procedure simulations for these types. In the material methodology of the novel Nuss procedure simulation, all tissues of the chest wall and metal bar were considered as only the elastic region of the material. However, more accurate simulation results can be obtained if precise material properties, such as the plastic region of the ICM, ductility/stiffness characteristics of the costal cartilage, and creep properties, are considered. When performing the novel Nuss procedure simulation for the severe type, the convergence of the analysis was slightly lower than that of the symmetric type. In addition, the computational time for the analysis was longer than that for the symmetric type, which reduced the efficiency of the study. Accordingly, studies that supplement measures, such as simplification of the 3D model of the chest wall or metal bar, and significant increases in computer performance are required. In the Nuss procedure, the metal bar is fastened to the ribs after rotation and equilibrium displacement. This fastening causes a physical impact on the chest wall and metal bar. In this study, fastening was not included due to the difficulty of simultaneously performing displacement and fastening of metal bars. Therefore, it is worth considering a Nuss procedure simulation study that includes fastening.

## 5 Conclusion

Patients with PEX were classified morphologically, and the novel Nuss procedure simulation was validated for the symmetrical, eccentric, and imbalanced types. The results of the simulations that applied the rotation and equilibrium displacement of the metal bar to each type of anterior sternal translation and HI closely mirrored the outcomes in actual patients. In particular, the symmetric and eccentric types yielded simulation results that closely resembled the actual results, significantly enhancing the credibility of the novel Nuss procedure simulation. By analyzing the mechanical physical quantities in the results of each type of simulation, the characteristic changes and morphological causes of the eccentricity and imbalance types that cannot be confirmed in the symmetrical type were predicted. Consequently, the precise predictive power of the novel Nuss procedure simulation was revealed by implementing the conflicting dynamic characteristics of each type through simulation. Therefore, it was demonstrated that the novel Nuss procedure simulation methodology can be fully applied to various types of severe PEX. However, a simplification strategy is required to facilitate novel Nuss procedure simulations for severe types.

This study compared the novel Nuss procedure simulation with actual surgery on patients of various morphological types. This is a validation method to reveal the accuracy of the simulation by comparing the simulation results with actual surgical results. If other validation studies, in addition to verification like this study, are published and stacked, the validity of the novel Nuss procedure simulation will increase. In these cases, thoracic surgeons can use the patient’s preoperative medical data to perform a novel Nuss procedure simulation during the surgical planning stage before performing the actual Nuss procedure. By considering patient-specific simulation results, thoracic surgeons can accurately and precisely plan chest wall correction goals and detailed surgical methods. Additionally, by predicting the surgical outcome through simulation, the virtual surgical outcome can be presented visually to the patient. In conclusion, the novel Nuss procedure simulation will be a means of delivering appropriate outcomes for thoracic surgeons and patients.

## Data Availability

The original contributions presented in the study are included in the article/Supplementary Material, further inquiries can be directed to the corresponding authors.
